# Intravenous administration of lidocaine directly acts on spinal dorsal horn and produces analgesic effect: An *in vivo* patch-clamp analysis

**DOI:** 10.1038/srep26253

**Published:** 2016-05-18

**Authors:** Miyuki Kurabe, Hidemasa Furue, Tatsuro Kohno

**Affiliations:** 1 Division of Anesthesiology, Niigata University Graduate School of Medical and Dental Sciences, 1-757 Asahimachi Dori, Chuo-Ku, Niigata City, 951-8510 Japan; 2 Department of Information Physiology, National Institute for Physiological Sciences, 5-1 Higashiyama, Myodaiji, Okazaki 444-8787, Japan

## Abstract

Intravenous lidocaine administration produces an analgesic effect in various pain states, such as neuropathic and acute pain, although the underlying mechanisms remains unclear. Here, we hypothesized that intravenous lidocaine acts on spinal cord neurons and induces analgesia in acute pain. We therefore examined the action of intravenous lidocaine in the spinal cord using the *in vivo* patch-clamp technique. We first investigated the effects of intravenous lidocaine using behavioural measures in rats. We then performed *in vivo* patch-clamp recording from spinal substantia gelatinosa (SG) neurons. Intravenous lidocaine had a dose-dependent analgesic effect on the withdrawal response to noxious mechanical stimuli. In the electrophysiological experiments, intravenous lidocaine inhibited the excitatory postsynaptic currents (EPSCs) evoked by noxious pinch stimuli. Intravenous lidocaine also decreased the frequency, but did not change the amplitude, of both spontaneous and miniature EPSCs. However, it did not affect inhibitory postsynaptic currents. Furthermore, intravenous lidocaine induced outward currents in SG neurons. Intravenous lidocaine inhibits glutamate release from presynaptic terminals in spinal SG neurons. Concomitantly, it hyperpolarizes postsynaptic neurons by shifting the membrane potential. This decrease in the excitability of spinal dorsal horn neurons may be a possible mechanism for the analgesic action of intravenous lidocaine in acute pain.

Intravenous administration of the local anaesthetic lidocaine has been used to treat neuropathic pain for several decades^1^ and significantly improves postoperative pain associated with complex spine surgery^2^ and cholecystectomy^3^. It is well established that lidocaine used for regional anaesthesia blocks impulses in peripheral nerves by inhibiting voltage-gated sodium (Na^+^) channels^4^. However, the underlying mechanisms of intravenous lidocaine may be more complex than simply the blockade of impulses in the nerve roots, because lidocaine has a remarkably broad pharmacological action.

Investigations of the optimum concentration of lidocaine for spinal and peripheral regional anaesthesia suggest that a high concentration (>200 μM) is required to block peripheral nerve fibre impulses^5^,^6^. The half maximal effective concentration of lidocaine for myelinated and unmyelinated dorsal root axons were 232 and 228 μM, respectively^6^. The half maximal inhibitory concentration for blocking different sciatic nerve fibres ranged from 320 to 800 μM^7^. However, when lidocaine is intravenously administered in doses from 1 to 5 mg/kg, its plasma concentration ranges from 4 to 20 μM. Therefore, the clinically effective plasma concentration of lidocaine to produce analgesia is far below that needed to block nerve impulses^8^,^9^.

In neuropathic or inflammatory pain animal models, intravenous lidocaine is thought to exert analgesic effects by blocking specific Na^+^ channels in injured nerves or dorsal root ganglia (DRG)^10^,^11^,^12^,^13^ because these channels are more sensitive to lidocaine^14^. The expression of tetrodotoxin (TTX)-sensitive Na^+^ channels, Nav1.3 and Nav1.7, is increased in the DRG or peripheral nerves after nerve injuries or inflammation, which causes hyperexcitability^14^,^15^,^16^. Several lines of evidence suggest that TTX-resistant channels expressed in nociceptors, Nav1.8 and Nav1.9, are especially important in neuropathic pain. However, the analgesic mechanisms of intravenous lidocaine in naïve rats with normal pain thresholds have not yet been examined. Although Na^+^ channels actions are undoubtedly the primary site of action for local anaesthetics, they are not necessarily the sole target of these drugs. Interactions with other signalling systems have been reported for many years, but have not received much attention, because the clinical importance of such effects has never been firmly established.

Multiple mechanisms regarding the site of action for the analgesic effects of lidocaine have been proposed, such as Na^+^ channel blockade in nerve fibres; interaction with many membrane receptors, proteins, and phospholipids; modulation of K^+^ channels, Ca^2+^ channels, *N*-methyl-D-aspartate (NMDA) receptors, α-amino-3-hydroxy-5-methyl-4-izoxazolepropionicacid (AMPA) receptors, and GTP-binding protein coupling receptors^17^,^18^ in dorsal horn neurons; direct action on the central nervous system including spinal cord neurons and the central terminals of DRG neurons; and the modulation of a balance between excitatory and inhibitory signalling in the spinal dorsal horn^19^,^20^,^21^,^22^,^23^,^24^,^25^,^26^. As the spinal dorsal horn, especially the substantia gelatinosa (SG), is a key area for pain processing^27^,^28^, we hypothesized that intravenous lidocaine acts on spinal SG neurons and modulates synaptic transmission. The *in vivo* patch-clamp technique is a useful tool to investigate changes in the balance between excitatory and inhibitory synaptic transmission in SG neurons because the neural circuit is preserved^28^. We therefore used this method to examine the mechanism of action of intravenous lidocaine in the spinal cord.

## Results

### Intravenous lidocaine has an analgesic effect on mechanical noxious response

**Figure 1:**
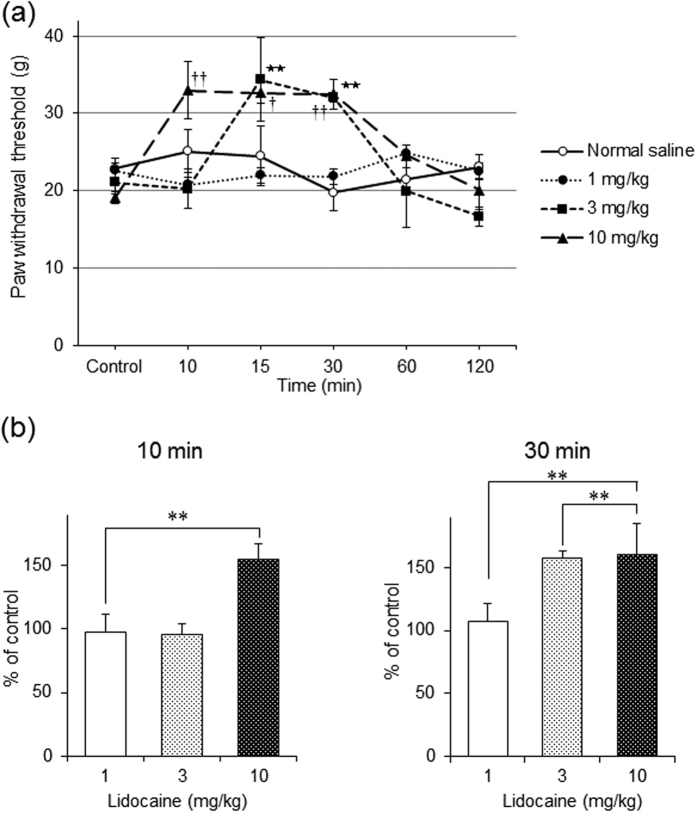
Assessment of the behavioural response to intravenous administration of lidocaine. (**a**) Intravenous lidocaine significantly increased the mechanical threshold for paw withdrawal in a dose-dependent manner. Concentrations of intravenous lidocaine ranged from 1 to 10 mg/kg. In each dose group, *n* = 6; **P < 0.01 in 3 mg/kg group and ^†^*P* < 0.05 and ^††^*P* < 0.01 in 10 mg/kg group compared to controls by one-way analysis of variance (ANOVA) with Tukey’s test, respectively. (**b**) Ten minutes after intravenous lidocaine administration, the mechanical threshold for paw withdrawal significantly increased in the 10 mg/kg group but not in the 3 mg/kg group. However, 30 min after lidocaine administration, the mechanical threshold significantly increased in both the 3 and 10 mg/kg groups. The mechanical threshold did not change at either time point in in the 1 mg/kg group. **P < 0.01 compared with controls by one-way ANOVA with Tukey’s test.

We used behavioural measures in rats to examine whether intravenous lidocaine has an analgesic effect on pain responses. The mechanical baseline withdrawal threshold was 20.3 ± 2.7 g (n = 24). Intravenous lidocaine significantly increased the mechanical threshold for paw withdrawal in a dose-dependent manner (each dose group; n = 6, *P* < 0.05 by one-way ANOVA, Fig. 1a).

Ten minutes after the intravenous administration of lidocaine, the mechanical threshold for paw withdrawal only significantly increased at a dose of 10 mg/kg (*P* < 0.01, by one-way ANOVA, Fig. 1b). However, 30 min after administration, the mechanical threshold significantly increased with doses of 3 and 10 mg/kg (both *P* < 0.01, by one-way ANOVA, Fig. 1b).

### Intravenous lidocaine decreases the spinal noxious response induced by peripheral pinch stimuli

We next investigated the effect of intravenous lidocaine on SG neuron responses to noxious stimulus using the *in vivo* whole-cell patch-clamp technique. Stable recordings were obtained from 157 SG neurons. All recorded neurons had resting membrane potentials lower than −50 mV. The average resting membrane potential and input membrane resistance were −62.2 ± 2.2 mV (*n* = 157) and 382.7 ± 30.1 MΩ (*n* = 157), respectively. All neurons exhibited excitatory postsynaptic currents (EPSCs) at a holding potential of −70 mV where no inhibitory postsynaptic currents (IPSCs) were observed. The average frequency and amplitude were 15.8 ± 3.8 Hz (*n* = 114) and 32.5 ± 5.1 pA (*n* = 114), respectively. These values were similar to those of a previously reported *in vivo* patch-clamp study^29^.

**Figure 2:**
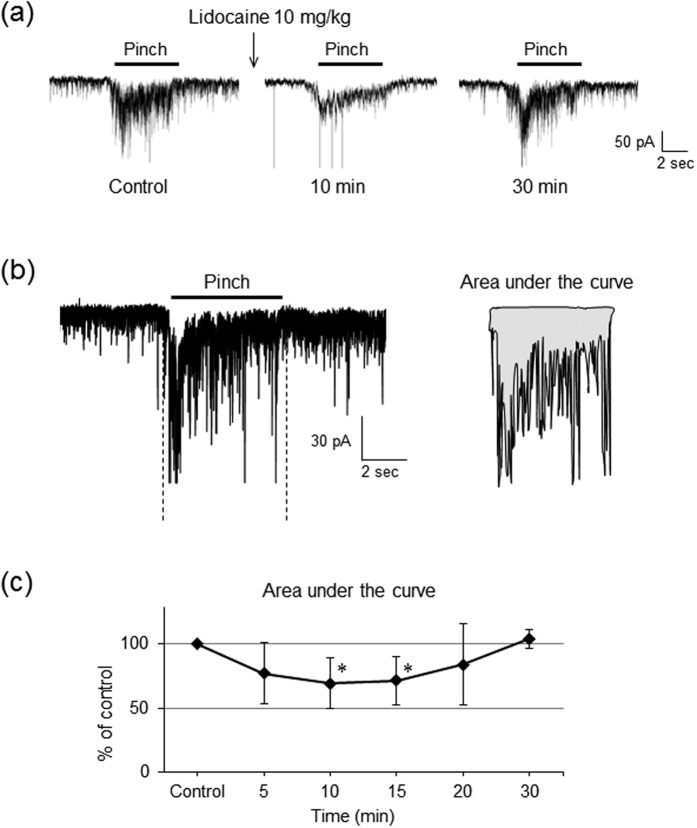
Intravenous administration of lidocaine decreases the noxious response induced by peripheral pinch stimuli. (**a**) Intravenous lidocaine (10 mg/kg) suppressed evoked excitatory postsynaptic currents (EPSCs) induced by peripheral pinch stimuli in a reversible manner. (**b**) Schematic diagrams showing areas under the curve. Analysis of the area surrounded by the baseline and border of EPSCs was performed with Clampfit10 software. (**b**) The area under the curve was significantly decreased by intravenous lidocaine (10 mg/kg) compared with controls both 10 and 15 min after administration. *n* = 12, *P < 0.05 compared to controls by Student’s paired *t*-test.

Pinch noxious stimuli applied to the ipsilateral hindlimb for 5 s evoked a barrage of EPSCs (Fig. 2a) with an increase in the area under the curve, surrounded by the baseline and border of EPSCs (Fig. 2b). Stimulating the contralateral hindlimb did not elicit any EPSCs. The area under the curve for the pinch responses was significantly reduced by intravenous lidocaine (10 mg/kg) compared with the control condition (n = 12, *P* < 0.05 by Student’s paired *t* test, Fig. 2a,c). The peak amplitudes were not determined because multiple summations resulting from the high frequency bursting of EPSCs made it difficult to obtain an accurate estimation.

### Intravenous lidocaine decreases the frequency of spontaneous EPSCs without changing their amplitude

**Figure 3:**
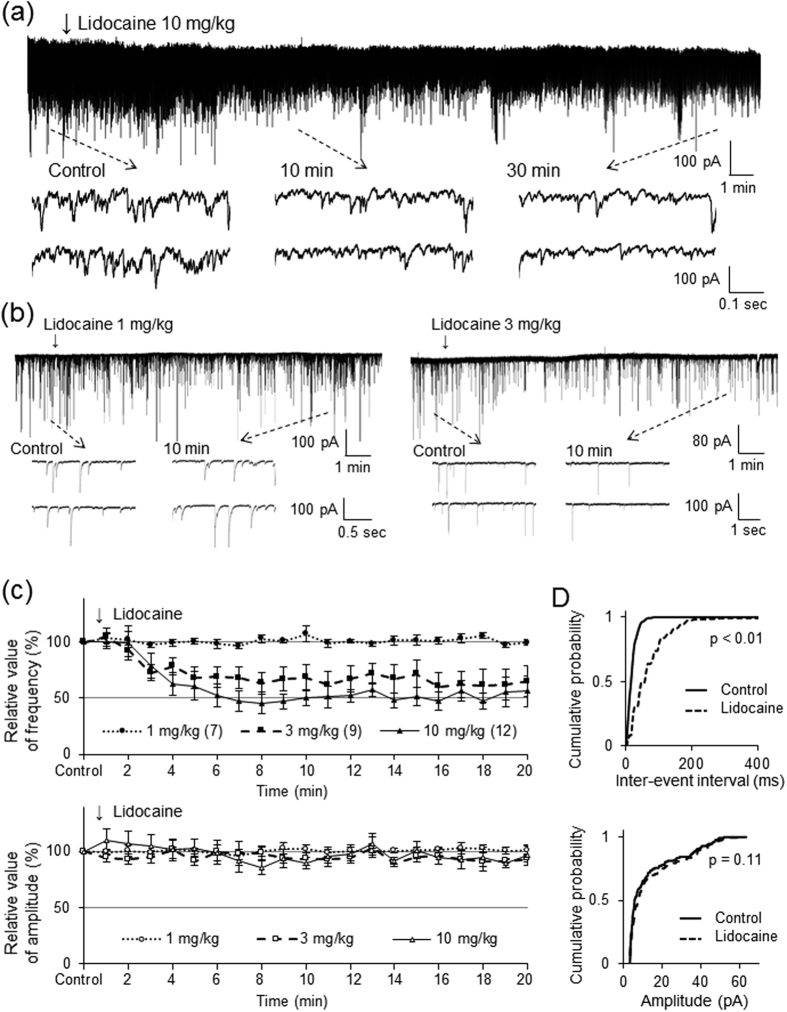
Intravenous administration of lidocaine decreases the frequency of spontaneous EPSCs but not change their amplitude. (**a**) Intravenous lidocaine (10 mg/kg) decreases the frequency of spontaneous excitatory postsynaptic currents (EPSCs). In contrast, it has no effect on the spontaneous EPSC amplitude. Downward arrows indicate outtakes of the top trace shown on an expanded timescale. Lidocaine was administered for 3 min from the arrowhead. (**b**) Intravenous lidocaine (3 mg/kg) decreases the frequency, but not the amplitude, of spontaneous EPSCs. In contrast, intravenous lidocaine (1 mg/kg) has no effect on the spontaneous EPSC frequency or amplitude. (**c**) Average frequency (upper graph) and amplitude (lower graph) of spontaneous EPSCs in the presence of lidocaine (10, 3, 1 mg/kg) relative to controls are shown as a function of time (mean ± standard error of the mean, n = 12, 9, 7, respectively). (**d**) Intravenous lidocaine (10 mg/kg) significantly shifts the cumulative distribution of the inter-event intervals to the right (*P* < 0.01, Kolmogorov-Smirnov test) (upper graph). In contrast, it has no effect on the cumulative distribution of the amplitudes (*P* = 0.11) (lower graph).

We next investigated the effect of intravenous lidocaine on spontaneous EPSCs to clarify the mechanisms underlying the inhibitory action of intravenous lidocaine on pinch-evoked EPSCs. Application of the α-amino-3-hydroxy-5-methl-4-isoxazole propionic acid (AMPA) receptor antagonist CNQX to the surface of the spinal cord rapidly and completely blocks spontaneous EPSCs^30^, indicating that they are mediated by glutamate release (data not shown). Intravenous lidocaine (10 mg/kg) decreased the frequency (47.1 ± 6.5% of controls, *P* < 0.01) but not the amplitude (93.7 ± 8.1% of controls, *P* = 0.46) of spontaneous EPSCs 10 min after administration (*n* = 12, Fig. 3a,c). Figure 3d shows the effects of intravenous lidocaine on the cumulative distributions of the spontaneous EPSC inter-event intervals and amplitudes before and after lidocaine application. When compared to the controls using the Kolmogorov-Smirnov test, intravenous lidocaine changed the cumulative frequency distribution of spontaneous EPSCs with significantly longer inter-event intervals (i.e., intravenous lidocaine decreased the frequency, *P* < 0.01). However, it had no effect on the cumulative amplitude distribution of the spontaneous EPSCs (*P* = 0.11).

The effect of intravenous lidocaine on spontaneous EPSCs varied in a dose-dependent manner. Although 3 mg/kg of intravenous lidocaine decreased the frequency (68.4% ± 11.5% of controls, *P* < 0.05), it had no effect on the amplitude (93.6 ± 12.4% of controls, *P* = 0.31) of spontaneous EPSCs 10 min after administration (*n* = 10, Fig. 3b,c). However, 1 mg/kg of intravenous lidocaine did not change the frequency (100.9 ± 1.6% of controls, *P* = 0.57) or amplitude (102.6 ± 4.9% of controls, *P* = 0.55) of spontaneous EPSCs 10 min after administration (*n* = 10, Fig. 3b,c).

### Intravenous lidocaine decreases the frequency of miniature EPSCs without changing their amplitude

**Figure 4:**
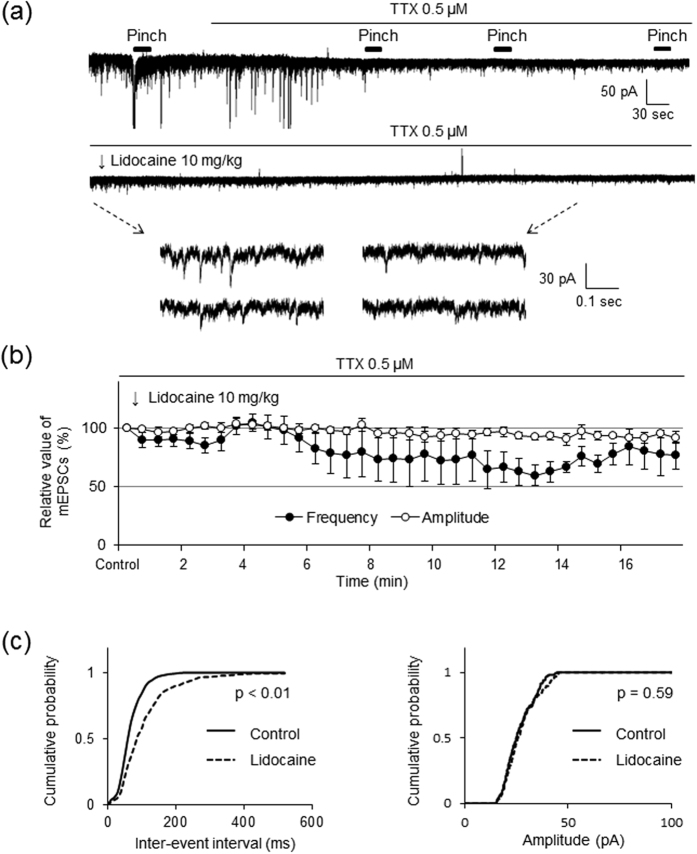
Intravenous administration of lidocaine decreases the frequency of miniature EPSCs without changing their amplitude. (**a**) Presented is an example of the effect of intravenous lidocaine on excitatory postsynaptic currents (EPSCs) in the presence of tetrodotoxin (TTX). After spinal superfusion of TTX (0.5 μM), the EPSCs elicited by peripheral pinch stimuli disappeared completely and large amplitudes of EPSCs were also inhibited. Intravenous lidocaine (10 mg/kg) reduced the frequency of miniature EPSCs. In contrast, intravenous lidocaine had no effect on the amplitude of miniature EPSCs. Downward arrows indicate outtakes of the top trace shown on an expanded timescale. Lidocaine was administered for 3 min from the arrowhead. (**b**) Average frequency and amplitude of miniature EPSCs in the presence of lidocaine (10 mg/kg) relative to controls are shown as a function of time (mean ± standard error of the mean, n = 10). (**c**) Intravenous lidocaine (10 mg/kg) significantly shifts the cumulative distribution of the inter-event intervals to the right (*P* < 0.01, Kolmogorov-Smirnov test) (left graph). In contrast, it has no effect on the cumulative distribution of the amplitudes (*P* = 0.59) (right graph).

To determine whether the site of the inhibitory action of intravenous lidocaine exists on the soma (postsynaptic recording neuron) or presynaptic terminals, we further investigated the effects of intravenous lidocaine on miniature EPSCs in the presence of the Na^+^ channel blocker TTX (0.5 μM). Since action potential conduction is blocked by TTX, the direct effects of intravenous lidocaine on the miniature release of glutamate from the presynaptic terminals can be isolated. As shown in Fig. 4a, superfusion of TTX completely abolished the evoked EPSCs induced by peripheral pinch stimuli and large amplitude EPSCs were also inhibited. In the presence of TTX, SG neurons still exhibited smaller amplitudes of EPSCs mediated by miniature glutamate release. Miniature EPSCs were also inhibited by intravenous lidocaine administration (see lower traces with an expanded time scale in Fig. 4a). Intravenous lidocaine (10 mg/kg) decreased the frequency (72.6 ± 13.4% of controls, *P* < 0.05) but not the amplitude (95.7 ± 6.4% of controls; *P* = 0.41) of miniature EPSCs 10 min after administration (*n* = 10, Fig. 4a,b).

Figure 4c shows the effects of intravenous lidocaine on the cumulative distributions of miniature EPSC inter-event intervals and amplitudes before and after lidocaine application. When compared to the controls using the Kolmogorov-Smirnov test, intravenous lidocaine changed the cumulative frequency distribution of miniature EPSCs with significantly longer inter-event intervals (i.e., intravenous lidocaine decreased the frequency, *P* < 0.01). However, it had no effect on the cumulative amplitude distribution of the miniature EPSCs (*P* = 0.59). These findings indicate that the intravenous lidocaine-induced decrease in glutamate release is presynaptic in origin.

### Intravenous lidocaine has no effect on spontaneous inhibitory postsynaptic currents

**Figure 5:**
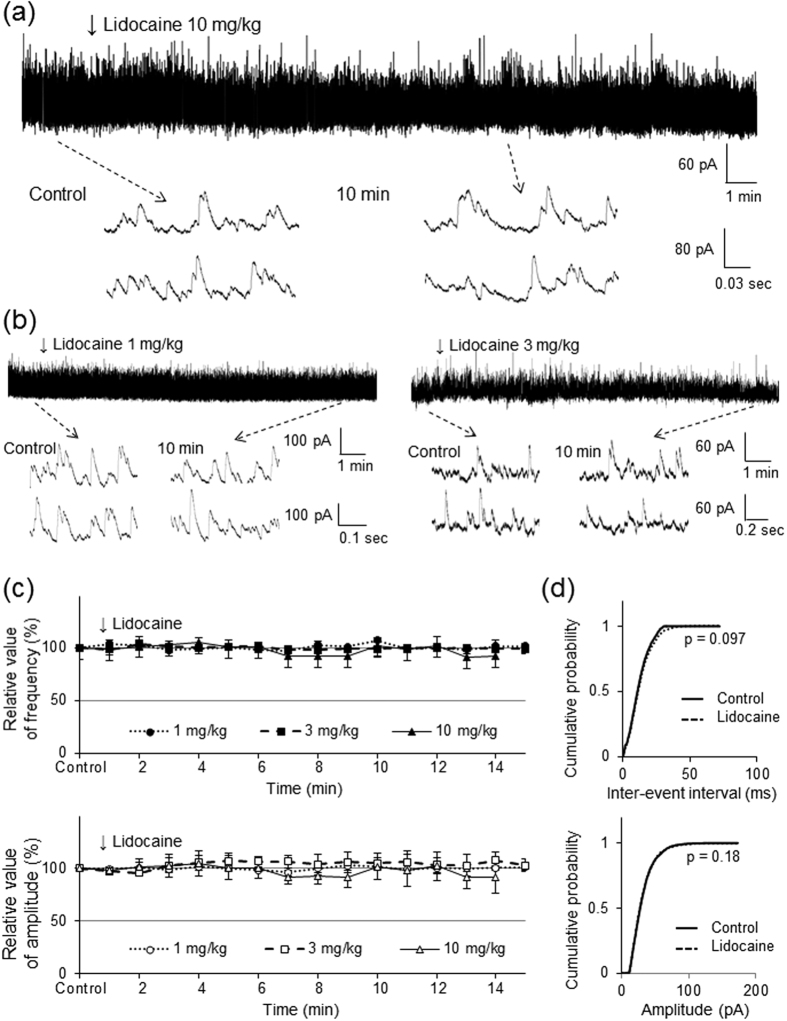
Intravenous administration of lidocaine has no effect on the spontaneous IPSC frequency or amplitude. (**a**) Spontaneous inhibitory postsynaptic currents (IPSCs) amplitude and frequency were unaffected by intravenous lidocaine (10 mg/kg). Downward arrows indicate outtakes of the top trace shown on an expanded timescale. Lidocaine was administered for 3 min from the arrowhead. (**b**) Spontaneous IPSC amplitude or frequency was unaffected by intravenous lidocaine (3 or 1 mg/kg). Downward arrows indicate outtakes of the top trace shown on an expanded timescale. Lidocaine was administered for 3 min from the arrowhead. (**c**) Average frequency (upper graph) and amplitude (lower graph) of spontaneous IPSCs in the presence of lidocaine (10, 3, 1 mg/kg) relative to controls are shown as a function of time (mean ± standard error of the mean; n = 7, 7, 4, respectively). (**d**) Intravenous lidocaine (10 mg/kg) has no effect on the cumulative distribution of the inter-event intervals (*P* = 0.097, Kolmogorov–Smirnov test) (upper graph) or the cumulative distribution of the amplitudes (*P* = 0.18) (lower graph).

We next examined the effect of intravenous lidocaine on spontaneous IPSCs elicited in SG neurons at a holding potential of 0 mV. All neurons exhibited spontaneous IPSCs at this holding potential whereas no EPSCs were observed as shown in our previous work^31^. The average frequency and amplitude were 42.4 ± 11.1 Hz (*n* = 12) and 38.3 ± 9.2 pA (*n* = 12), respectively. These values were similar to those of a previously reported *in vivo* patch-clamp study^29^. Intravenous lidocaine (10 mg/kg) did not change the frequency (101.8 ± 9.3% of controls, *P* = 0.2) or amplitude (101.1 ± 19.7% of controls, *P* = 0.38) of spontaneous IPSCs 10 min after administration (*n* = 7, Fig. 5a,c). Figure 5d shows the effects of intravenous lidocaine on the cumulative distributions of spontaneous IPSC inter-event intervals and amplitudes before and after lidocaine application. When compared to the controls using the Kolmogorov-Smirnov test, intravenous lidocaine had no effect on the cumulative inter-event interval (*P* = 0.097) or amplitude distribution of spontaneous IPSCs (*P* = 0.18, Fig. 5d).

Low doses of intravenous lidocaine also did not change the frequency (3 mg/kg, 98.8 ± 3.3% of controls, *n* = 7, *P* = 0.24; 1 mg/kg, 99.8 ± 4.3% of controls, *n* = 4, *P* = 0.47) or amplitude (3 mg/kg, 104.8 ± 5.8% of controls, *n* = 7, *P* = 0.19; 1 mg/kg, 105.2 ± 7.4% of controls, *n* = 4, *P* = 0.13) of spontaneous IPSCs 10 min after administration (Fig. 5b,c).

We also investigated the effect of intravenous lidocaine on miniature IPSCs in the presence of TTX. Intravenous lidocaine (10 mg/kg) did not change the frequency (95.5 ± 13.2% of controls, *P* = 0.27) or amplitude (95.7 ± 6.0% of controls, *P* = 0.12) of miniature IPSCs (*n* = 5, data not shown). These results suggest that intravenous lidocaine has no effect on inhibitory synaptic transmission in the spinal dorsal horn.

### Intravenous lidocaine produces an outward current in SG neurons

**Figure 6:**
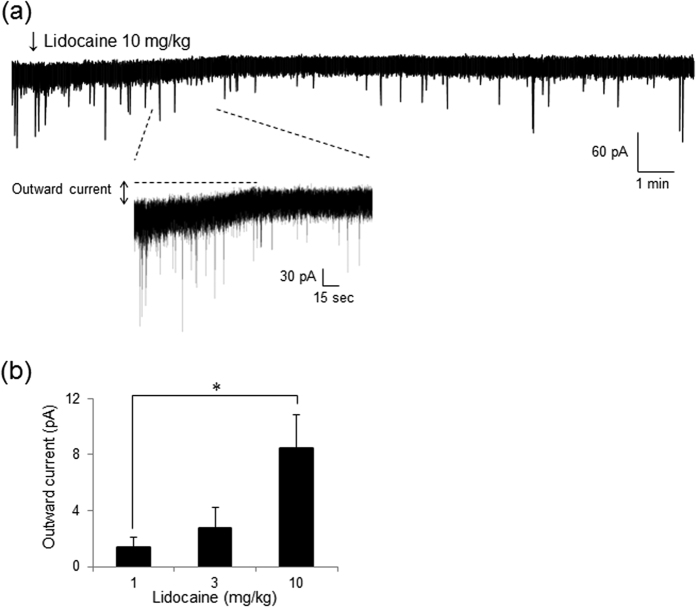
Intravenous administration of lidocaine produces an outward current in SG neurons. (**a**) Intravenous lidocaine (10 mg/kg) produces an outward current at a holding potential of −70 mV. Traces below are shown on an expanded time scale. Lidocaine was administered for 3 min from the arrowhead. (**b**) Intravenous lidocaine induces an outward current in SG neurons. The average amplitude of the intravenous lidocaine (10 mg/kg)-induced outward current was larger than that of lidocaine (1 mg/kg). *P < 0.05 compared to 1 mg/kg group by one-way analysis of variance (ANOVA).

Intravenous lidocaine dose-dependently induced an outward current in SG neurons (Fig. 6). The average amplitude of the intravenous lidocaine (10 mg/kg)-induced outward current was 8.5 ± 2.1 pA (*P* < 0.05 compared to the 1 mg/kg group) in 8 of 12 (66.7%) neurons. Low doses of intravenous lidocaine produced similar responses: 2.7 ± 1.1 pA (*P* = 0.45 compared to 1 mg/kg group) with 3 mg/kg in 4 of 9 (44.4%) neurons, and 1.4 ± 0.8 pA with 1 mg/kg in 3 of 7 (42.9%) neurons. The lidocaine (10 mg/kg)-induced outward currents were confirmed from 3 to 10 minutes after administration of lidocaine and persisted for more than 30 minutes. Furthermore, in the presence of TTX (0.5 μM), intravenous lidocaine (10 mg/kg) also induced an outward current (12.1 ± 5.7 pA) in 7 of 10 (70%) neurons (data not shown). These results suggest that intravenous lidocaine produces a postsynaptic outward current to hyperpolarize SG neurons in the spinal dorsal horn.

## Discussion

We demonstrated that intravenously administered lidocaine produces analgesic effects on the withdrawal response to mechanical noxious stimuli at doses of 3 and 10 mg/kg. To clarify the mechanism underlying its analgesic effect, we investigated the effect of lidocaine on subthreshold synaptic transmission and postsynaptic membrane currents in SG neurons of the superficial dorsal horn, which has an important role in transmitting and modulating nociceptive information^27^,^28^. Our *in vivo* patch-clamp technique enabled us to quantitatively analyse the spinal actions of intravenous lidocaine and we found that lidocaine at the same doses used in the behavioural analysis inhibited spontaneous and pinch-evoked excitatory synaptic responses without affecting inhibitory synaptic responses in SG neurons. Miniature analysis of the synaptic events suggests that lidocaine acts on presynaptic terminals to reduce glutamate release. Furthermore, intravenous lidocaine also produced outward (hyperpolarizing) currents. The effective doses for the subthreshold spinal neuronal responses were approximately one order of magnitude lower than those for blocking impulses in peripheral afferent nerve fibres in previous reports^5^,^6^. This is the first study to show the subthreshold and multiple direct actions of intravenous lidocaine in the spinal cord.

### Intravenous lidocaine has an analgesic effect on mechanical and acute noxious responses in naïve rats

Previous studies demonstrated that intravenous lidocaine has a minimal effect on pain thresholds in acute pain^32^,^33^. Abram *et al.* showed that intravenous lidocaine (3 mg bolus + 25 μg/min infusion) had no effect on acute pain (formalin phase 1 flinching activity) but significant reduced chronic pain (formalin phase 2 flinching activity)^32^. On the other hand, intravenous lidocaine (5, 10, and 15 mg/kg) suppressed the maintained formalin-evoked activity (phases 1 and 2) *in situ* from single sural nerve fibres^33^. These studies evaluated pain behaviour by assessing responses to thermal stimulation such as latency to licking or flinching^32^,^34^ or withdrawal responses using von Frey filaments^35^. We used a dynamic aesthesiometer to evaluate the effects of intravenous lidocaine on mechanical withdrawal threshold to show that lidocaine significantly and dose-dependently increased the mechanical thresholds in naïve rats. This suggests that intravenous lidocaine also has an analgesic effect on acute noxious transmission.

### Intravenous lidocaine inhibits excitatory synaptic transmission in spinal dorsal horn neurons

To elucidate the spinal actions of intravenous administration of lidocaine, we investigated excitatory synaptic responses mediated by glutamate release^30^ in SG neurons *in vivo*, which exhibited spontaneous EPSCs with an average amplitude and frequency of 33 pA and 16 Hz, respectively. The amplitude was almost comparable to that of EPSCs evoked by cutaneous noxious pinch stimulation (see control response in Fig. 2a). Application of a voltage-gated Na^+^ channel blocker inhibited large amplitudes of spontaneous EPSCs (Fig. 4a). These results suggest that SG neurons *in vivo* receive excitatory glutamatergic synaptic inputs evoked by spontaneous neuronal firing. Intravenous lidocaine decreased the frequency of spontaneous EPSCs by 47% at 10 mg/kg and 68% at 3 mg/kg and shifted the cumulative frequency distribution toward significantly longer inter-event intervals (Fig. 3). This implies that lidocaine reduces the frequency of excitatory glutamatergic inputs, which would regulate the basal excitability of SG neurons. However, lidocaine did not change the amplitude of spontaneous EPSCs or the cumulative amplitude distribution, suggesting that lidocaine has no action on postsynaptic glutamate receptor activity. In support of this, lidocaine did not affect the amplitude of miniature EPSCs in the presence of TTX. On the contrary, lidocaine decreased miniature EPSC frequency, suggesting that it also acts on glutamate release from the presynaptic terminals of SG neurons.

What type of mechanism would decrease EPSC frequency? A previous report shows that NMDA- and AMPA-induced extracellular signal-regulated kinase activation was suppressed by lidocaine and may be one of the mechanisms through which lidocaine prevents surgical pain^18^. Furthermore, intravenous lidocaine-induced analgesic action in rats subjected to the formalin test for acute pain was partly reversed by D-serine, a full agonist at the glycine-binding site of glutamate NMDA receptors^34^. Liu *et al.* showed that local anaesthetics (*e.g.*, bupivacaine and ropivacaine) inhibited high-voltage-activated Ca^2+^ currents evoked in dorsal horn neurons. Because the half-maximal blockade of Ca^2+^ channels occurred at higher concentrations compared with Na^+^ channels (~5–15 times)^36^, their blockade may not contribute to interrupting neurotransmission at physiologically relevant concentrations of intravenous lidocaine. Although the precise mechanisms are unclear, our findings suggest that intravenous lidocaine acts directly on excitatory glutamatergic synaptic inputs to SG neurons to reduce glutamate release.

### Intravenous lidocaine has no effect on inhibitory transmission in spinal dorsal horn neurons

Given that intravenous lidocaine facilitates a descending inhibitory system^37^,^38^ or local inhibitory actions^39^, we can expect effects of intravenous lidocaine on inhibitory synaptic transmission in SG neurons. Glycine is one of the main inhibitory neurotransmitters in the central nervous system^40^ and is involved in the development of neuropathic pain^41^. A study reported that the analgesic action of intravenous lidocaine was reduced by intrathecally administered strychnine (an antagonist at inhibitory glycine-receptors) in the chronic constriction injury model using rats, suggesting a glycine-like action of lidocaine or its metabolites in the spinal cord^34^. However, our results showed that intravenous lidocaine had no effect on spontaneous or miniature IPSCs. Although previous studies have suggested that intravenous lidocaine may modulate such an inhibitory system, our findings show that intravenous lidocaine does not modify basal inhibitory synaptic transmission in SG neurons.

### Intravenous lidocaine acts on postsynaptic SG neurons in the spinal cord

Furthermore, we found that intravenous lidocaine induced outward currents in SG neurons. These results suggest that intravenous lidocaine acts on postsynaptic neurons to hyperpolarize the membrane and therefore shift the potential even further from the firing threshold, thus reducing excitability. One possibility is that intravenous lidocaine facilitates a descending inhibitory system and increases the release of noradrenaline or serotonin, which induces outward currents. Noradrenaline and serotonin cause membrane hyperpolarization by opening K^+^ channels in dorsal horn neurons^42^,^43^. However, in the present study, the K^+^ channel blocker caesium was present in the pipette solution^42^,^43^. In addition, our results showing that intravenous lidocaine had no effect on IPSCs suggest that it did not facilitate the release of gamma-aminobutyric acid (GABA) and/or glycine that activate local inhibitory neurons and induce outward currents. Moreover, our finding that in the presence of TTX, intravenous lidocaine also induced an outward current suggests that intravenous lidocaine directly binds to postsynaptic SG neurons. As local anaesthetics could also interact with membrane phospholipids and proteins including various receptors (e.g., NMDA receptors^44^, Ca^2+^ channels^36^, and G protein-coupled receptors^45^), inhibition of the activities at these receptors or channels may contribute to the occurrence of outward currents.

### Physiological significance of intravenous lidocaine-induced modulation of synaptic transmission in the spinal cord

Na^+^ channels are a key factor in regulating neuronal excitability and generating action potentials. Several lines of evidence suggest that the expression of TTX-sensitive and TTX-resistant Na^+^ channels is altered in nociceptors following nerve injury or inflammation. Therefore, decreased ectopic activity originating from the injured peripheral nerves or DRG by blockade of these Na^+^ channels may be a possible analgesic mechanism for intravenous lidocaine to influence neuropathic or inflammatory pain^10^,^11^,^12^,^13^. However, in the present study, there is no change in Na^+^ channel expression because we used naïve rats. We also examined the effect of intravenous lidocaine on acute pain. The blood concentration of intravenous lidocaine is not sufficient to block TTX-resistant and TTX-sensitive Na^+^ channels in naïve rats^32^,^46^, thus, Na^+^ channels are unlikely to be the target of intravenous lidocaine with regard to its analgesic effect on acute pain in naïve rats.

Interestingly, the action of intravenous lidocaine on the mechanical stimuli in the behavioural test had a longer duration than that in pinch-evoked EPSCs in the electrophysiological experiments. Likewise, the action of lidocaine was longer on the spontaneous and miniature EPSCs and outward current. Some investigators have reported that the suppressive effect of lidocaine on action potentials in extracellular recording had a significantly shorter duration (less than 30 min) than that in behavioural experiments (more than a few hours)^10^,^24^. There are numerous spontaneous neuronal inputs in spinal dorsal horn neurons without input from peripheral nerves. However, only action potentials are observed in extracellular recording. Therefore, the main analgesic effect of intravenous lidocaine with a long duration will likely be due to decreased spontaneous excitatory inputs from presynaptic terminals. We found prolonged pre- and postsynaptic effects of lidocaine associated with behavioural experiments with an extended duration.

The present study analysed the effect of intravenous lidocaine on acute pain in naïve rats. Intravenous lidocaine has been used clinically to relieve neuropathic or postoperative pain. Because the physiological characteristics and mechanisms of these animal pain models may vary from those of naïve rats, the effects of intravenous lidocaine may likewise differ. The *in vivo* patch-clamp method is a good tool to investigate changes in the balance between excitatory and inhibitory synaptic transmission in spinal cord neurons because the neural circuit is preserved. Further study with this method is required to clarify the mechanism of action of intravenous lidocaine on neuropathic and inflammatory pain.

In summary, we found that intravenous lidocaine produces analgesic effects on acute pain in naïve rats. To elucidate its mechanism, we investigated the effect of lidocaine on synaptic transmission that is “below the threshold” of the action potential using the *in vivo* patch-clamp technique. Our results suggest that intravenous lidocaine inhibits glutamate synaptic transmission in the spinal cord and acts on postsynaptic SG neurons for a longer duration than previously reported. This direct action of intravenous lidocaine on spinal SG neurons reveals its important role in the alleviation of acute pain.

## Materials and Methods

### Animals

All animal experiments were conducted in accordance with international guidelines on the ethical use of animals, and all efforts were made to minimize animal pain or discomfort. Animal housing and surgical procedures were approved by the Institutional Animal Care and Use Committee of Niigata University Graduate School of Medical and Dental Science. Male Wistar rats (6 to 10 weeks) weighing 200 to 380 g were housed in plastic cages at 22 ± 2 °C on a standard 12-h light/dark cycle with food and water available *ad libitum*.

### Behavioural tests

Rats were acclimated to the experimental room for at least 1 h before lidocaine administration. Rats were placed on a perforated metal platform and mechanical stimuli were delivered to the plantar surface of the hind paw using the Dynamic Plantar Aesthesiometer (37450, Ugo Basile, Comerio, Italy) positioned beneath the platform. The equipment raises a straight metal 0.5-mm diameter filament until it touches the plantar surface of the hindpaw and exerts an increasing upward force (from 1 to 50 g over 20 s) until the paw is withdrawn or the preset cut-off is reached (50 g). Study of the dose-dependent effects of lidocaine involved four groups (1, 3, 10 mg/kg of lidocaine and normal saline), each with *n* = 6 animals. Lidocaine or normal saline (100 μl per animal) was administered intravenously through a tail vein. The mechanical withdrawal threshold was measured for the right paw from the average of 5 aesthesiometer trials at 10, 15, 30, 60, and 120 min after lidocaine administration.

### *In vivo* patch-clamp recording from SG neurons

The methods used for *in vivo* patch-clamp recording from the SG neurons were similar to those described previously^29^,^47^,^48^. Briefly, male Wistar rats were anesthetized with urethane (1.2–1.5 g/kg, intraperitoneally) and the tail artery and vein were cannulated for blood pressure monitoring and drug administration, respectively. If a withdrawal reflex appeared, then a supplemental dose of urethane was given during surgery and the data collection period. A heating pad was placed beneath the rat to maintain its body temperature. Thoracolumbar laminectomy was performed at the level of T12 to L2 to expose the lumbar enlargement of the spinal cord. The rat was placed in a stereotaxic apparatus (Model ST-7; Narishige, Tokyo, Japan). After the dura mater was opened, the pia-arachnoid membrane was removed to make a window large enough to allow the patch electrode to enter the spinal cord. The surface of the spinal cord was continuously perfused with Krebs solution equilibrated with 95% O_2_ and 5% CO_2_ gas mixture at a flow rate of 10 to 15 ml/min maintained at 36 °C using a temperature controller (TC-324B; Warner Instruments, Hamden, CT, USA). The Krebs solution contained (in mM) 117 NaCl, 3.6 KCl, 2.5 CaCl_2_, 1.2 mgCl_2_, 1.2 NaH_2_PO_4_, 25 NaHCO_3_, and 11 D-glucose.

The patch electrodes were pulled from borosilicate glass capillaries (OD, 1.5 mm) using a puller (p-97; Sutter Instruments, Novato, CA, USA). The resistance of a typical patch pipette was 4–8 MΩ when filled with internal solution. The patch pipette solution contained (in mM) 110 Cs_2_SO_4_, 5 tetraethylammonium, 0.5 CaCl_2_, 2 mgCl_2_, 5 EGTA, 5 HEPES, and 5 ATP-Mg (pH 7.2). The electrode was advanced into the spinal dorsal horn through the window in the pia-arachnoid membrane using a micromanipulator (Model MWS-32S; Narishige, Tokyo, Japan). A giga-ohm seal (resistance > 5 GΩ) was then formed with neurons at a depth of 50–150 μm from the spinal cord surface. After making a giga-ohm seal, the patch membrane was ruptured by a brief period of negative pressure, resulting in a whole-cell configuration. Signals were collected using an Axopatch 200B amplifier in conjunction with a Digidata 1440A A/D converter (Molecular Devices, Sunnyvale, CA, USA) and stored on a personal computer using the pCLAMP 10 data acquisition program (Molecular Devices). The data were analysed using Mini Analysis 6.0 software (Synaptosoft, Fort Lee, NJ, USA) or pCLAMP 10. All experiments were performed in voltage-clamp mode at a holding potential of −70 or 0 mV.

### Drug application

Lidocaine was dissolved in 0.9% saline and adjusted to a volume of 100 μl in each concentration group. It was intravenously administered via a tail vein catheter for 3 min during *in vivo* patch-clamp recordings. For spinal application, drugs were diluted in Krebs solution and superfused onto the spinal cord without altering the perfusion rate or temperature. The time necessary for the solution to flow from the stopcock to the spinal cord surface was approximately 10 s. Lidocaine hydrochloride was purchased from Sigma-Aldrich (St. Louis, MO, USA) and 6-cyano-7-nitroquinoxaline-2, 3-dione (CNQX) and tetrodotoxin (TTX) from Wako (Osaka, Japan).

### Stimulation protocols

We shaved fur from the rat hindquarter (the lumbar, gluteal, and hindlimb regions) to exclude the effect of non-noxious touch stimuli to the hair. In voltage-clamp mode, we identified a neuron responding exclusively to noxious pinch stimuli. We marked the area on the skin where a noxious pinch stimulus produced the neural response. The noxious mechanical stimuli were then applied to the marked site with toothed forceps. To maintain a fixed strength of noxious stimulation, the toothed forceps were clamped during skin pinching for a constant duration of 5 s to include pinching pain.

### Study approval

All animal experiments were conducted in accordance with international guidelines on the ethical use of animals and all efforts were made to minimize the amount of pain or discomfort experienced by the animals. Animal housing and surgical procedures were approved by the Institutional Animal Care and Use Committee of Niigata University Graduate School of Medical and Dental Science (Approval No. 342-5).

### Statistical analysis

All numerical data are shown as mean ± S.E.M. Statistical significance was determined as *P* < 0.05. Student’s paired *t*-tests, one-way analyses of variance (ANOVAs), Tukey’s multiple comparisons, or Kolmogorov-Smirnov tests were used as indicated for statistical analyses. In the behavioural and electrophysiological data, n refers to the number of neurons studied.

## Additional Information

**How to cite this article**: Kurabe, M. *et al.* Intravenous administration of lidocaine directly acts on spinal dorsal horn and produces analgesic effect: An *in vivo* patch-clamp analysis. *Sci. Rep.* **6**, 26253; doi: 10.1038/srep26253 (2016).

## References

[b1] Koppert W., Ostermeier N., Sittl R., Weidner C. & Schmelz M. Low-dose lidocaine reduces secondary hyperalgesia by a central mode of action. Pain 85, 217–224 (2000). 10692621 10.1016/s0304-3959(99)00268-7

[b2] Farag E. *et al.* Effect of Perioperative Intravenous Lidocaine Administration on Pain, Opioid Consumption, and Quality of Life after Complex Spine Surgery. Anesthesiology 119, 932–940 (2013). 23681143 10.1097/ALN.0b013e318297d4a5

[b3] Cassuto J., Wallin G., Hogstrom S., Faxen A. & Rimback G. Inhibition of postoperative pain by continuous low-dose intravenous infusion of lidocaine. Anesthesia and analgesia 64, 971–974 (1985). 3898920

[b4] Catterall W. A. From ionic currents to molecular mechanisms: the structure and function of voltage-gated sodium channels. Neuron 26, 13–25 (2000). 10798388 10.1016/s0896-6273(00)81133-2

[b5] Jaffe R. A. & Rowe M. A. Subanesthetic concentrations of lidocaine selectively inhibit a nociceptive response in the isolated rat spinal cord. Pain 60, 167–174 (1995). 7784102 10.1016/0304-3959(94)00117-W

[b6] Jaffe R. A. & Rowe M. A. Differential nerve block. Direct measurements on individual myelinated and unmyelinated dorsal root axons. Anesthesiology 84, 1455–1464 (1996). 8669687 10.1097/00000542-199606000-00022

[b7] Huang J. H., Thalhammer J. G., Raymond S. A. & Strichartz G. R. Susceptibility to lidocaine of impulses in different somatosensory afferent fibers of rat sciatic nerve. J Pharmacol Exp Ther 282, 802–811 (1997). 9262344

[b8] Challapalli V., Tremont-Lukats I. W., McNicol E. D., Lau J. & Carr D. B. Systemic administration of local anesthetic agents to relieve neuropathic pain. Cochrane Database Syst Rev, 10.1002/14651858.CD003345.pub2 (2005). PMC6483498 16235318

[b9] Tanelian D. L. & MacIver M. B. Analgesic concentrations of lidocaine suppress tonic A-delta and C fiber discharges produced by acute injury. Anesthesiology 74, 934–936 (1991). 2021210 10.1097/00000542-199105000-00020

[b10] Devor M., Wall P. D. & Catalan N. Systemic lidocaine silences ectopic neuroma and DRG discharge without blocking nerve conduction. Pain 48, 261–268 (1992). 1589245 10.1016/0304-3959(92)90067-L

[b11] Chabal C., Russell L. C. & Burchiel K. J. The effect of intravenous lidocaine, tocainide, and mexiletine on spontaneously active fibers originating in rat sciatic neuromas. Pain 38, 333–338 (1989). 2510116 10.1016/0304-3959(89)90220-0

[b12] Chevrier P., Vijayaragavan K. & Chahine M. Differential modulation of Nav1.7 and Nav1.8 peripheral nerve sodium channels by the local anesthetic lidocaine. Br J Pharmacol 142, 576–584 (2004). 15148257 10.1038/sj.bjp.0705796 PMC1574965

[b13] Leffler A., Reiprich A., Mohapatra D. P. & Nau C. Use-dependent block by lidocaine but not amitriptyline is more pronounced in tetrodotoxin (TTX)-Resistant Nav1.8 than in TTX-sensitive Na^+^ channels. J Pharmacol Exp Ther 320, 354–364 (2007). 17005919 10.1124/jpet.106.109025

[b14] Amir R. *et al.* The Role of Sodium Channels in Chronic Inflammatory and Neuropathic Pain. The Journal of Pain 7, S1–S29 (2006). 16632328 10.1016/j.jpain.2006.01.444

[b15] Black J. A. *et al.* Upregulation of a silent sodium channel after peripheral, but not central, nerve injury in DRG neurons. Journal of neurophysiology 82, 2776–2785 (1999). 10561444 10.1152/jn.1999.82.5.2776

[b16] Lindia J. A., Kohler M. G., Martin W. J. & Abbadie C. Relationship between sodium channel NaV1.3 expression and neuropathic pain behavior in rats. Pain 117, 145–153 (2005). 16061326 10.1016/j.pain.2005.05.027

[b17] Yanagidate F. & Strichartz G. R. Local anesthetics. Handbook of experimental pharmacology, Vol. 177 (eds Stein C.) Part II 95–127 (Springer Berlin Heidelberg, 2007). 17087121 10.1007/978-3-540-33823-9_4

[b18] Zhang L. *et al.* Different effects of local anesthetics on extracellular signal-regulated kinase phosphorylation in rat dorsal horn neurons. European journal of pharmacology 734, 132–136 (2014). 24726558 10.1016/j.ejphar.2014.03.048

[b19] Komai H. & McDowell T. S. Local anesthetic inhibition of voltage-activated potassium currents in rat dorsal root ganglion neurons. Anesthesiology 94, 1089–1095 (2001). 11465602 10.1097/00000542-200106000-00025

[b20] Xiong Z. & Strichartz G. R. Inhibition by local anesthetics of Ca^2+^ channels in rat anterior pituitary cells. European journal of pharmacology 363, 81–90 (1998). 9877085 10.1016/s0014-2999(98)00769-9

[b21] Hollmann M. W. *et al.* Inhibition of m3 muscarinic acetylcholine receptors by local anaesthetics. Br J Pharmacol 133, 207–216 (2001). 11325812 10.1038/sj.bjp.0704040 PMC1572757

[b22] Bach F. W., Jensen T. S., Kastrup J., Stigsby B. & Dejgard A. The effect of intravenous lidocaine on nociceptive processing in diabetic neuropathy. Pain 40, 29–34 (1990). 2339012 10.1016/0304-3959(90)91047-M

[b23] Woolf C. J. & Wiesenfeld-Hallin Z. The systemic administration of local anaesthetics produces a selective depression of C-afferent fibre evoked activity in the spinal cord. Pain 23, 361–374 (1985). 3937116 10.1016/0304-3959(85)90006-5

[b24] Sotgiu M. L., Biella G., Castagna A., Lacerenza M. & Marchettini P. Different time-courses of i.v. lidocaine effect on ganglionic and spinal units in neuropathic rats. Neuroreport 5, 873–876 (1994). 8061286 10.1097/00001756-199404000-00005

[b25] Dohi S. *et al.* An analgesic action of intranvenously administered lidocaine on dorsal-horn neurons responding to noxious thermal stimulation. Anesthesiology 51, 123–126 (1979). 222173 10.1097/00000542-197908000-00006

[b26] Kuner R. Central mechanisms of pathological pain. Nat Med 16, 1258–1266 (2010). 20948531 10.1038/nm.2231

[b27] Kohno T., Moore K. A., Baba H. & Woolf C. J. Peripheral nerve injury alters excitatory synaptic transmission in lamina II of the rat dorsal horn. J Physiol 548, 131–138 (2003). 12576493 10.1113/jphysiol.2002.036186 PMC2342789

[b28] Furue H., Narikawa K., Kumamoto E. & Yoshimura M. Responsiveness of rat substantia gelatinosa neurones to mechanical but not thermal stimuli revealed by *in vivo* patch-clamp recording. J Physiol 521 Pt 2, 529–535 (1999). 10581321 10.1111/j.1469-7793.1999.00529.x PMC2269671

[b29] Funai Y. *et al.* Systemic dexmedetomidine augments inhibitory synaptic transmission in the superficial dorsal horn through activation of descending noradrenergic control: an *in vivo* patch-clamp analysis of analgesic mechanisms. Pain 155, 617–628 (2014). 24355412 10.1016/j.pain.2013.12.018 PMC4237836

[b30] Furue H., Katafuchi T. & Yoshimura M. Sensory processing and functional reorganization of sensory transmission under pathological conditions in the spinal dorsal horn. Neuroscience Research 48, 361–368 (2004). 15041189 10.1016/j.neures.2003.12.005

[b31] Hiroshi Baba M. D. PhD, Koki Shimoji M. D. PhD & Megumu YOshimura M. D. Ph.D. Norepinephrine facilitates inhibitory transmission in substantia gelatinosa of adult rat spinal cord (part1) Effects on axon terminals of GABAergic andGlycinergic neurons. Anesthesiology 92, 473–484 (2000). 10691235 10.1097/00000542-200002000-00030

[b32] Abram S. E. & Yaksh T. L. Systemic lidocaine blocks nerve injury-induced hyperalgesia and nociceptor-driven spinal sensitization in the rat. Anesthesiology 80, 383–391; discussion 325A (1994). 8311320

[b33] Puig S. & Sorkin L. S. Formalin-evoked activity in identified primary afferent fibers: systemic lidocaine suppresses phase-2 activity. Pain 64, 345–355 (1996). 8740613 10.1016/0304-3959(95)00121-2

[b34] Muth-Selbach U. *et al.* Antinociceptive effects of systemic lidocaine: involvement of the spinal glycinergic system. European journal of pharmacology 613, 68–73 (2009). 19394327 10.1016/j.ejphar.2009.04.043

[b35] Sinnott C. J., Garfield J. M. & Strichartz G. R. Differential efficacy of intravenous lidocaine in alleviating ipsilateral versus contralateral neuropathic pain in the rat. Pain 80, 521–531 (1999). 10342413 10.1016/S0304-3959(98)00245-0

[b36] Scholz A. Mechanisms of (local) anaesthetics on voltage-gated sodium and other ion channels. Br J Anaesth 89, 52–61 (2002). 12173241 10.1093/bja/aef163

[b37] Klas S. & PAbelson A. U. H. Intravenously administred lidocaine in therapeutic doses increases the intraspinal release of acetylcholine in rats. Neuroscience Letters 317, 93–96 (2002). 11755248 10.1016/s0304-3940(01)02440-5

[b38] Lauretti G. R. Mechanisms of analgesia of intravenous lidocaine. Rev Bras Anestesiol 58, 280–286 (2008). 19378524 10.1590/s0034-70942008000300011

[b39] Biella G. & Sotgiu M. L. Central effects of systemic lidocaine mediated by glycine spinal receptors: an iontophoretic study in the rat spinal cord. Brain Res 603, 201–206 (1993). 8096422 10.1016/0006-8993(93)91238-n

[b40] Legendre P. The glycinergic inhibitory synapse. Cell Mol Life Sci 58, 760–793 (2001). 11437237 10.1007/PL00000899 PMC11337367

[b41] Dohi T., Morita K., Kitayama T., Motoyama N. & Morioka N. Glycine transporter inhibitors as a novel drug discovery strategy for neuropathic pain. Pharmacol Ther 123, 54–79 (2009). 19393690 10.1016/j.pharmthera.2009.03.018

[b42] Yoshimura M. & Furue H. Mechanisms for the anti-nociceptive actions of the descending noradrenergic and serotonergic systems in the spinal cord. J Pharmacol Sci 101, 107–117 (2006). 16766858 10.1254/jphs.crj06008x

[b43] Abe K. *et al.* Responses to 5-HT in morphologically identified neurons in the rat substantia gelatinosa *in vitro*. Neuroscience 159, 316–324 (2009). 19141313 10.1016/j.neuroscience.2008.12.021

[b44] Furutani K., Ikoma M., Ishii H., Baba H. & Kohno T. Bupivacaine inhibits glutamatergic transmission in spinal dorsal horn neurons. Anesthesiology 112, 138–143 (2010). 20032703 10.1097/01.anes.0000365964.97138.9a

[b45] Hollmann M. W., Fischer L. G., Byford A. M. & Durieux M. E. Local anesthetic inhibition of m1 muscarinic acetylcholine signaling. Anesthesiology 93, 497–509 (2000). 10910501 10.1097/00000542-200008000-00030

[b46] Luo Z. *et al.* The effect of intravenous lidocaine on brain activation during non-noxious and acute noxious stimulation of the forepaw: a functional magnetic resonance imaging study in the rat. Anesth Analg 108, 334–344 (2009). 19095870 10.1213/ane.0b013e31818e0d34 PMC2681082

[b47] Furue H. *In vivo* blind patch-clamp recording technique. In Patch Clamp Techniques 171–182 (Springer, 2012).

[b48] Georgiev S. K., Furue H., Baba H. & Kohno T. Xenon inhibits excitatory but not inhibitory transmission in rat spinal cord dorsal horn neurons. Mol Pain 6, 25 (2010). 20444263 10.1186/1744-8069-6-25 PMC2873505

