# Corrigendum: Intravenous administration of lidocaine directly acts on spinal dorsal horn and produces analgesic effect: An *in vivo* patch-clamp analysis

**DOI:** 10.1038/srep46814

**Published:** 2017-06-01

**Authors:** Miyuki Kurabe, Hidemasa Furue, Tatsuro Kohno

Scientific Reports 6: Article number: 26253; 10.1038/srep26253 published online: 05 18 2016; updated: 06 01 2017.

The Author Contributions statement in this Article is incomplete, where:

“M.K. and T.K. designed the experiments; M.K. conducted experiments and analysed the data; H.F. and T.K. wrote the manuscript. All authors reviewed the manuscript”.

should read:

“M.K. and T.K. designed the experiments; M.K. conducted experiments and analysed the data; M.K., H.F. and T.K. wrote the manuscript. All authors reviewed the manuscript”.

